# Supplementary material for: Patterns of Intron Gain and Loss in Fungi
Source: PLoS Biol. 2004 Nov 30;2(12):e422. doi: 10.1371/journal.pbio.0020422 (PMC532390; doi:10.1371/journal.pbio.0020422)
Supplement: Table S1 — Also available at http://genes.mit.edu/NielsenEtAl/. (4.3 MB ZIP). [file pbio.0020422.st001.zip › NielsenEtAl/html/1207.html]

AN7736.1.NCU02147.1.MG03061.1.FG08845.1


```
 CLUSTAL W (1.82) Multiple Sequence Alignments - Introns Inserted


Sequence 1: NCU02147.1	659 aa
Sequence 2: FG08845.1	632 aa
Sequence 3: MG03061.1	656 aa
Sequence 4: AN7736.1	637 aa
Alignment Length: 683 aa
Number Identitical Residues: 252 aa
Alignment Score (without introns) 13160


MG03061.1 	MPPQ--EDALLRRPLYV~YDLPPEVLSSLALKAGADASDEPETETSIPSESRPQTPESTG
NCU02147.1	MASK--QEDLQRRPVYL~YDIDADILNTVALKSDADITAEPVNET------AKQTIDTTA
FG08845.1 	MAKT--NEDLLRRPLYL1YDLPVNVLDTLVLKDDADAEDIAAAES------AASSLTESS
AN7736.1  	MTTKNQVEELLKRPLYV1YDLPQELLASIALKGEDQPITVEDAEP-------TRKESSDD
          	*.  .. : * :**:*: **:  ::* ::.**   :       *.         .     

MG03061.1 	TSSTLVGSQA-CSLCGLSFDGLEEQRSHLKSDWHHYNLKQKLRGLKPVSESEFEKLIGDL
NCU02147.1	SEPTASGSQASCSLCSLKFDNVQEQRSHLKTDFHHYNLKQKLNGLSPVTEAKFEELVNNL
FG08845.1 	SDSNLVGTQA-CSLCGLTFTTVIDQRGHLKSDLHHYNLKQKLRGQKPVSEAEFEKLVGNL
AN7736.1  	VQEHAIATSTSCAICKVSFANVQEQREHVRSDHHKYNVRAQLRGNAVLDEVQFTKAIGEL
          	 .    .:.::*::* :.*  : :** *:::* *:**:: :*.*   : * :* : :.:*

MG03061.1 	DESLSGEDDSSEDEDEQAKGDST--LVTLLKKQANIVDKSRKDKSVEEERETSKNPRGSG
NCU02147.1	DESISGSDSSDSEDEEEGQDKDTNMLSALLKKQANIVEKMTKEGGDDGENDGEKVKSSNG
FG08845.1 	DESLSGSDSEESDEEEDGRQEST--LTTLLKKQARLTENAGDD--DEGETAG---RAGRG
AN7736.1  	DESISGSESS---EEEDGDAGDQ--LSALLKRQAKISQAAEEG----KESAT---PRGVG
          	***:**.:..   ::*:.   .   * :***:**.: :   .      *        . *

MG03061.1 	TPPLVWFTSPQLPENHYYGIYRAIFTNEELATEDKILDAVKSRQLDPISMPKPLTNKDGT
NCU02147.1	SQPLLWFSSPKLPENSYYGVYKAMFTPEELKKEDAIMDAIKSRQLAPITMGKPPKDAN--
FG08845.1 	KPPLIWFSSPLLPEKNYFGMYRAILTGDEQRNQD-LVEVLKKKQVEPIAMPK-IKEGA--
AN7736.1  	KHPLLWFTHPALPPNTSLGVYRALFTNEEQEERKCLVDSLKKKQLAPIYPQQ--KDNG--
          	. **:**: * ** :   *:*:*::* :*   .. ::: :*.:*: **   :  .:    

MG03061.1 	MGPSAATYTGRHIFMCMIGGGHFAAMVVSLAPKKSKSSAP--LNREATVLAHKTFHRYTT
NCU02147.1	--DIPPNYTGKHIFMCMIGGGHFAAMVVSLNPRKTKHGTTGPLNKEAVVLVHKTFHRYTT
FG08845.1 	--LPEVAYKGPHIFLCMIGGGHFAAMVVSLAPRPNKNGTT--MNREATVLAHKTFHRYTT
AN7736.1  	----QPATPSPHIFMCMIGGGHFAAMLVSLAPEVHRKQGG-VEERQARVIAHKSFHRYTT
          	         . ***:***********:*** *.  :       :::* *:.**:******

MG03061.1 	RRKQGGSQSANDNAKGTAHSAGSSLRRYNEQALVEDVRNLLSDWKGLLDTADLLFVRATG
NCU02147.1	RRKQGGSQSANDNAKGAAHSAGANLRRYNEQALVEDVRGLLAEWKGLIDTSDLLFIRATG
FG08845.1 	RRKQGGSQSANDNAKGAAHSAGSSLRRYNEQALVEDVRNLLQEWKALIDTSELLFIRATG
AN7736.1  	RRKQGGSQSANDAAKGAAHSAGSSLRRYNEATLEKEIRELLQDWGKMINEAQLLFVRAAG
          	************ ***:*****:.****** :* :::* ** :*  ::: ::***:**:*

MG03061.1 	VTNRRTLFGPYEGQVLRANDPRLRGFPFSTKRATQNELMRSFIELTRLKVREILP---VF
NCU02147.1	LTNRRTLFGPYDGQVLRANDPRIRGFPFMTRRATQNELMRAFIELTRLKVRQIVP---EA
FG08845.1 	MTNRRTLFGPYEGQVLKHNDTRLRGFPFSTRRATQNELMRSFIELTRLKVREIVP---VE
AN7736.1  	STNRRVLFGQHEGQVLKQNDPRLRGFPFSTRRATQTELMRCFKELTRVKISQVDEAALAA
          	 ****.*** ::****: **.*:***** *:****.****.* ****:*: ::  ::   

MG03061.1 	EQPPQPEQKQAPGPKQ--TPVKKLSEEEETAQLHTQQMQALIRRSKLPSLLTYIKNNNLT
NCU02147.1	AAPTTESPKRPKSPKPAAASKPKLSEEEETLLLHTQQIQALVKRSKVPALLSYLSTNDIS
FG08845.1 	ETKKEADVVPKTSTKP---SKPKLSEEEETALLHTSQLQAFVRRSKVPALLSYLKNNSIT
AN7736.1  	AATKQREESKPSTPKPQ-PQKPKISKEDEAAIMHTTQIQALIRRSKIPALMSYLSKNSIP
          	      .      .*   .   *:*:*:*:  :** *:**:::***:*:*::*:..*.:.

MG03061.1 	PDFRFHPPETQQNHHAPTPLHLAASLNSATVAHGLLVRGGANPTIHNGQGRTAFELAGDR
NCU02147.1	LK-EFRFFP--ADHHTPTPLHLAASQNSAPLVTGLLTRAGADPTIPNGDGRTAFELAGDR
FG08845.1 	ADFEFQPVE--QNHHAPRPLHLAAAQNAAPLVLGLLVRGGADPTIKNNEGKTPFELAGER
AN7736.1  	SSFTFQPSDVQQNFRCPTPLHLASNLNSPAMVLALLTKLDADPTATNAEGRTPFELAGDR
          	 .  *:    . :.: * *****:  *:..:. .**.: .*:**  * :*:*.*****:*

MG03061.1 	ATRDAFRVARAELGEDKWTWGEDGAKVPAALTRAEADQREERE----AKKEADRRKAEEE
NCU02147.1	ATRDAFRVARSELGEEAWDW--EAAGVPVALSRDEALKRTEREKNEAKEKEDQRRKAEEE
FG08845.1 	STRDAFRVARSELGEAKWAW--DDAKVPPAMTKAEADKRDEREKKEACDKEAERRKAEEE
AN7736.1  	ATRDAFRVARHELGESKWNW--EAANIPPAISKAEADNRLQRERKTAEEEEANRRKAEMD
          	:********* ****  * *  : * :* *::: ** :* :** . : .:* :***** :

MG03061.1 	RLKREEAASGAKKGGASGARKAGG-LLDGTVPKTAQDRREEEMRGLTPEQKMRIERERRA
NCU02147.1	RLKKE----GPKVDATKNGKKAGGSVLAAAMKLTPQERREEEAKGLTPEMKMRLERERRA
FG08845.1 	RLQSE----GPKVPE---GRKQKGNILAAGLKQTPQEKREAEARGLTPEMRMRLERERRA
AN7736.1  	RLRQE-------EAATAARMTKKGGRTVGSVEKTAQERREEETRGMTPEMRMRLERERRA
          	**: *          :    .  *.   . :  *.*::** * :*:*** :**:******

MG03061.1 	RAAEERLKRMQSGS~----------
NCU02147.1	RAAEERIRRLQGGQ2VYQCQSVANV
FG08845.1 	RAAEERLRKMQSGG~----------
AN7736.1  	RAAEERIRRMQQGQ~KQ--------
          	******::::* *
```
